# The combined effect of lifestyle factors and polygenic scores on age at onset in Parkinson’s disease

**DOI:** 10.1038/s41598-024-65640-x

**Published:** 2024-06-25

**Authors:** Carolin Gabbert, Leonie Blöbaum, Theresa Lüth, Inke R. König, Amke Caliebe, Sebastian Sendel, Björn-Hergen Laabs, Christine Klein, Joanne Trinh

**Affiliations:** 1 Institute of Neurogenetics, University of Lübeck, Ratzeburger Allee 160, 23538 Lübeck, Germany; 2 Institute of Medical Biometry and Statistics, University of Lübeck, Lübeck, Germany; 3 Institute of Medical Informatics and Statistics, Kiel University, University Hospital Schleswig-Holstein, Kiel, Germany

**Keywords:** Caffeine, Smoking, NSAID, Gene-environment, *GBA1*, Genotype, Risk factors, Parkinson's disease

## Abstract

The objective of this study was to investigate the association between a Parkinson’s disease (PD)-specific polygenic score (PGS) and protective lifestyle factors on age at onset (AAO) in PD. We included data from 4367 patients with idiopathic PD, 159 patients with *GBA1*-PD, and 3090 healthy controls of European ancestry from AMP-PD, PPMI, and Fox Insight cohorts. The association between PGS and lifestyle factors on AAO was assessed with linear and Cox proportional hazards models. The PGS showed a negative association with AAO (*β* = − 1.07, *p* = 6 × 10^–7^) in patients with idiopathic PD. The use of one, two, or three of the protective lifestyle factors showed a reduction in the hazard ratio by 21% (*p* = 0.0001), 44% (*p* < 2 × 10^–16^), and 55% (*p* < 2 × 10^–16^), compared to no use. An additive effect of aspirin (*β* = 7.62, *p* = 9 × 10^–7^) and PGS (*β* = − 1.58, *p* = 0.0149) was found for AAO without an interaction (*p* = 0.9993) in the linear regressions, and similar effects were seen for tobacco. In contrast, no association between aspirin intake and AAO was found in *GBA1*-PD (*p* > 0.05). In our cohort, coffee, tobacco, aspirin, and PGS are independent predictors of PD AAO. Additionally, lifestyle factors seem to have a greater influence on AAO than common genetic risk variants with aspirin presenting the largest effect.

## Introduction

Parkinson’s disease (PD) is a complex neurodegenerative disorder. Besides monogenic forms of PD that explain about 5% of PD cases^1^, GWAS studies have shown that idiopathic PD is highly polygenic^2,3^. The largest meta-GWAS of PD to date identified 90 independent risk loci across 78 genomic regions that explained between 16 and 36% of the heritable risk of PD^2^. That study additionally determined the proportion of SNP-based heritability explained by their PD GWAS and found their 1805 variant polygenic score (PGS) to explain about 26% of PD heritability^2^. The calculation of PGSs provides the opportunity to summarize the effect of the heritable risk to develop the disease on the individual level. Several studies already evaluated the association of PGSs for PD and affection status, age at onset (AAO), or PD-related symptoms^4–13^.

In addition to common genetic risk factors, environmental and lifestyle factors have consistently shown an association with PD susceptibility. While some environmental and lifestyle factors, e.g., pesticides, heavy metals, type 2 diabetes mellitus, and traumatic brain injuries, have been reported to increase the risk for PD^14–19^, there are also several environmental and lifestyle factors, e.g., smoking, coffee and black tea, non-steroidal anti-inflammatory drugs (NSAIDs), and physical activity, that have been frequently described as protective regarding the risk for PD, AAO and symptom progression^20–31^. We have previously investigated the effect of environmental and lifestyle factors on the AAO in PD in the Fox Insight cohort and found a protective effect for coffee drinking, tobacco use, and aspirin intake, while no or only a marginal difference in AAO was found for black tea drinking, ibuprofen, and other NSAIDs^32^. Interactions between genetic modifiers and lifestyle factors can further affect PD risk. Gene-environment interactions have been shown between the genetic assembly and a patient’s lifestyle. Thus far, there are known interactions between *GRIN2A*, *ADORA2A*, and *CYP1A2* and coffee^33,34^ as well as between *RXRA*, *SLC17A6*, and *HLA-DRB1* and smoking^35,36^. In contrast, studies investigating the effect of environmental and lifestyle factors or gene-environment interactions on PD AAO are only sparse. While some studies found a protective effect of coffee and smoking on PD AAO^32,37–44^, literature on the effect of aspirin on AAO is lacking^32^. It also remains unclear how gene-environment interactions or a genetic predisposition to PD risk together with the presence of certain lifestyle factors influences the AAO in PD.

Herein, we examine AAO associations of the PGS and the combined effect of the established protective lifestyle factors coffee drinking, tobacco use, and aspirin intake in PD. Our rationale in selecting for these three factors lies in (1) the robustness for previous findings on PD risk; (2) our own findings that coffee drinking, tobacco use, and aspirin intake is associated with later AAO, while no association with AAO was found for black tea in this study group; and (3) the access and availability of this particular data across several datasets. We investigate whether coffee drinking, tobacco use, and aspirin intake are positively associated with the AAO in PD and if these lifestyle factors further have an additive or interactive effect with respect to the PGS on PD AAO.

## Materials and methods

### Study demographics

Three datasets containing genetic, environmental, and lifestyle data from the Accelerating Medicine Partnership Parkinson’s Disease Knowledge Platform (AMP-PD), the Parkinson’s Progression Markers Initiative (PPMI), and the Fox Insight cohort were included in this study. The complete information on the data harmonization of AMP-PD cohorts comprises of eight sub-cohorts in total (BioFIND, HBS, LBD, LCC, PDBP, PPMI, STEADY-PD3 and SURE-PD3). The Fox Investigation for New Discovery of Biomarkers in Parkinson's Disease (BioFIND) is a cross‐sectional, multicenter biomarker study designed to discover and verify biomarkers in clinically typical PD^45^. The Harvard Biomarker Study (HBS) is a large biobank that recruits patients with early-stage PD or mild cognitive impairment to discover new targets for drugs, new genes, and new diagnostics^46^. The LBD Study (International Lewy Body Genomics Consortium) performed whole-genome sequencing in large cohorts of Lewy body dementia cases and neurologically healthy controls to study the genetic architecture of this disease and to generate a resource for the scientific community^47^. The LRRK2 Cohort Consortium (LCC) was created to assemble and investigate groups of people with and without PD who carry mutations in the *LRRK2* gene. The National Institute of Neurological Disorders and Stroke (NINDS) Parkinson's Disease Biomarkers Program (PDBP) aims to accelerate the discovery of promising new diagnostic and progression biomarkers for PD. The Parkinson’s Progression Markers Initiative (PPMI) is a longitudinal observational study designed to establish biomarker-defined cohorts and identify clinical, imaging, genetic, and biospecimen PD progression markers to accelerate disease-modifying therapeutic trials^48^. The NINDS funded STEADY-PD III trial (STEADY-PD3) is a Phase 3, parallel group, placebo-controlled study evaluating the efficacy of isradipine 10 mg daily as a disease-modifying agent in early PD for 36 months^49^. The Study of URate Elevation in Parkinson's Disease, phase 3 study (SURE-PD3) is a randomized, double-blind, placebo-controlled trial of urate-elevating inosine treatment to slow clinical decline in early PD. The AMP-PD data received from all sub-cohorts was centrally harmonized, curated, quality controlled, and consolidated into one dataset using both automated and manual approaches, which included the aligning of variables between datasets, decoding of numeric coded variables, clean-up and standardization of medication names, diagnosis, level of education, and the alignment of visit names between cohorts.

The Fox Insight data facilitates discovery, validation, and reproducibility in Parkinson's disease research^50^. The dataset is generated through routine longitudinal assessments (health and medical questionnaires), one-time questionnaires about environmental exposure and healthcare preferences, and genetic data collection. Patient recruitment details for the Fox Insight study have been previously described^50^. Volunteers for the Fox Insight study were recruited through digital channels (e.g., social network ads, search engine marketing, and email newsletters) and on-the-ground recruitment efforts (e.g., research events, clinician referrals). All Fox Insight participants were 18 years of age or older and provided informed consent. Upon registration, participants were divided into patients with PD and healthy controls, whereas the latter were asked about new diagnoses every three months. PD patients responded to health, non-motor assessments, motor assessments, quality of life, and lifestyle questionnaires through twenty questionnaires that are part of routine longitudinal assessments. Detailed questions about lifestyle, personal habits, living and work environments, medication and healthy history are provided in the Environmental Exposure Questionnaires (PD-RFQ-U). The Fox Insight and AMP-PD cohorts are all established data resources from the Michael J. Fox Foundation. Ethical approval was obtained from the Ethics Committee of the University of Lübeck. Patients and healthy controls from PPMI are included in the AMP-PD cohort for genome sequencing and more detailed lifestyle data was documented as part of the PPMI cohort. Information on known genetic mutations were provided by the cohort platforms in the clinical demographics, which included mutation carrier status for LRRK2 p.G2019S and p.R1441G, GBA p.N409S, and SNCA p.A53T in AMP-PD, as well as LRRK2 p.G2019S and GBA p.R535H, p.N409S, and p.E365K mutation carrier status in Fox Insight. As these are some of the most common genetic causes and common genetic risk variants for PD, these were provided by the cohort platforms. Information on other mutations were not included on the platforms. Known mutation carriers were excluded from the group of patients with idiopathic PD and the healthy controls. No other exclusion criteria were applied. In total, 7616 unrelated participants were included in our study: 4367 patients with PD, without a known genetic cause of PD, 159 patients with variants in *GBA1,* which harbor some of the strongest genetic risk variants in PD, and 3090 healthy controls (Table 1). In this study group, the mean AAO of patients with PD without a known genetic cause of PD was 60.5 years (standard deviation, SD =  ± 9.7 years, range: 19.3–89.1 years) and the mean age at examination (AAE) was 64.7 years (SD =  ± 9.0 years, range: 33.0–91.5 years). Of the patients with PD, 2480 (56.8%) were men and 1887 (43.2%) were women. Of the 4367 patients with PD, 1986 were from the AMP-PD cohort, of which 386 were from the PPMI subgroup of AMP-PD, and 2381 were from the Fox Insight cohort.

**Table 1:** Demographics of the study group.

	Patients with PD^a^	Patients with *GBA1*-PD	Healthy controls
N_total_	4367	159	3090
Male/female (%)	2480/1887 (56.8%/43.2%)	78/81 (49.1%/50.9%)	1495/1595 (48.4%/51.6%)
Coffee drinkers/non-drinkers (%)	1914/607 (75.9%/24.1%)	118/27 (81.4%/18.6%)	65/11 (85.5%/14.5%)
Tobacco users/non-users (%)	1468/2563 (36.4%/63.6%)	54/95 (36.2%/63.8%)	310/368 (45.7%/54.3%)
Aspirin users/non-users (%)	863/1658 (34.2%/65.8%)	28/64 (30.4%/69.6%)	29/47 (38.2%/61.8%)
Mean AAO (SD, range)	60.5 (9.7, 19.3–89.1)	61.9 (9.5, 28.5–83.9)	NA
Mean AAE (SD, range)	64.7 (9.0, 33.0–91.5)	65.7 (8.9, 32.0–86.1)	69.9 (13.0, 16.0–90.0)
N_AMP-PD_	1986	NA	3090
Male/female (%)	1258/728 (63.3%/36.7%)	NA	1495/1595 (48.4%/51.6%)
Coffee drinkers/non-drinkers (%)	103/37 (73.6%/26.4%)	NA	65/11 (85.5%/14.5%)
Tobacco users/non-users (%)	663/987 (40.2%/59.8%)	NA	310/368 (45.7%/54.3%)
Aspirin users/non-users (%)	56/84 (40.0%/60.0%)	NA	29/47 (38.2%/61.8%)
Mean AAO (SD, range)	60.8 (9.9, 28.0–89.0)	NA	NA
Mean AAE (SD, range)	64.5 (9.5, 33.0–90.0)	NA	69.9 (13.0, 16.0–90.0)
N_PPMI_^b^	386	NA	200
Male/female (%)	251/135 (65.0%/35.0%)	NA	133/67 (66.5%/33.5%)
Coffee drinkers/non-drinkers (%)	103/37 (73.6%/26.4%)	NA	65/11 (85.5%/14.5%)
Tobacco users/non-users (%)	46/94 (32.9%/67.1%)	NA	31/45 (40.8%/59.2%)
Aspirin users/non-users (%)	56/84 (40.0%/60.0%)	NA	29/47 (38.2%/61.8%)
Mean AAO (SD, range)	61.5 (9.4, 35.0–85.0)	NA	NA
Mean AAE (SD, range)	62.0 (9.5, 35.0–85.5)	NA	61.6 (10.6, 31.0–83.0)
N_Fox Insight_	2381	159	NA
Male/female (%)	1222/1159 (51.3%/48.7%)	78/81 (49.1%/50.9%)	NA
Coffee drinkers/non-drinkers (%)	1811/570 (76.1%/23.9%)	118/27 (81.4%/18.6%)	NA
Tobacco users/non-users (%)	805/1576 (33.8%/66.2%)	54/95 (36.2%/63.8%)	NA
Aspirin users/non-users (%)	807/1574 (33.9%/66.1%)	28/64 (30.4%/69.6%)	NA
Mean AAO (SD, range)	60.3 (9.5, 19.3–89.1)	61.9 (9.5, 28.5–83.9)	NA
Mean AAE (SD, range)	64.9 (8.7, 33.0–91.5)	65.7 (8.9, 32.0–86.1)	NA

*PD* Parkinson’s disease; *AAO* age at onset; *AAE* age at examination; *SD* standard deviation.

^a^Patients with a known genetic cause of PD were excluded.

^b^Samples from the PPMI sub-cohort are already included in AMP-PD and only lifestyle data was added.

The group of patients with *GBA1*-PD, who carried one of the GBA1 variants p.R535H (NM_000157.4, c.1604G > A), p.N409S (NM_000157.4, c.1226A > G), and p.E365K (NM_000157.4, c.1093G > A) consisted of 159 patients with a mean AAO of 61.9 years (SD =  ± 9.5 years, range: 28.5–83.9 years) and a mean AAE of 65.7 years (SD =  ± 8.9 years, range: 32.0–86.1 years). Of these, 78 (49.1%) were men and 81 (50.9%) were women.

The group of healthy controls consisted of 3090 participants with a mean AAE of 69.9 years (SD =  ± 13.0 years, range: 16.0–90.0 years). While 1495 (48.4%) of the controls were men, 1595 (51.6%) were women. All participants included in this study were of white European ancestry as reported in the participant summaries.

### Genetic data and polygenic score estimate

AMP-PD genetic data contained whole-genome sequencing (WGS) data from six unified cohorts (BioFIND, HBS, PDBP, PPMI, SURE-PD3, LBD)^51^. All samples of the AMP-PD dataset were processed by the TOPMed Freeze 9 Variant Calling Pipeline for joint genotyping^51^. The genetic dataset from AMP-PD was stored in a binary PLINK format^52^. The dataset was filtered using PLINK 1.9 according to standard quality control filtering steps, excluding SNPs with a minor allele frequency < 0.01, a missingness per sample > 0.02, a missingness per SNP > 0.05, and that failed Hardy–Weinberg equilibrium at a threshold of 1 × 10^–50^.

For the PGS calculation, a previously proposed composition of 1805 variants associated with PD risk^2,4^ was used together with the reference alleles and effect sizes. In our study sample, 1725 of the PGS SNPs were included in the AMP-PD data, with additional 13 SNPs that were represented by proxy SNPs. Proxy SNPs were evaluated with *SNiPA*^53^ and had to be in a linkage disequilibrium of > 0.98 with the SNP of interest. In total 1738 SNPs were used for the PGS calculation with the PLINK *score* function. The PGS values were subsequently standardized by subtraction of the mean and division by the standard deviation of the PGS among controls^4^. This standardized PGS was used for all further analyses. Density plots were created with the base-R function *density* and receiver operating characteristic (ROC) curves and corresponding areas under the curve (AUCs) were calculated with the R package *pROC* (R version 4.3.0)^54,55^.

In order to perform a principal component analysis (PCA), the unfiltered genetic dataset from AMP-PD was pruned based on the linkage disequilibrium (LD) using an r^2^ threshold of 0.3 with the PLINK 1.9 *indep-pairwise* function. Subsequently, the dataset was filtered for a minor allele frequency > 0.3 and a genotyping rate > 0.99. The PCA was performed using PLINK 1.9 *pca* function.

### Lifestyle and environmental data

Available environmental and lifestyle data was harmonized across the unified cohorts in AMP-PD. For this, available clinical assessment data was curated and transformed by aligning variable names from AMP-PD studies to a global mapping file. The harmonization further included simplifying the information on caffeine consumption and use of tobacco, resulting in the indication whether a subject in AMP-PD had ever used caffeinated beverages or tobacco. Therefore, more detailed environmental and lifestyle data was also obtained from the PPMI sub-cohort separately. PPMI FOUND (Follow up of persons with Neurologic Disease) uses the Parkinson’s Disease Risk Factor Questionnaires (PD-RFQ), which collect life-long information on lifestyle and health, including habits, occupation and residence. The Risk Factor Questionnaire was developed by the National Institute of Environmental Health Sciences PD-RFQ Epidemiology Working Group of the Collaborative Centers for Parkinson’s Disease Environmental Research and provides a standard assessment tool for general use in epidemiologic studies of PD (https://www.commondataelements.ninds.nih.gov/report-viewer/23723/Risk%20Factor%20Questionnaire%20(RFQ-U)). It has been validated for self-report and interview. In PPMI, the information from the PD-RFQs is captured by telephone or other remote consultation methods. The PPMI FOUND data included detailed information on the consumption of coffee, tobacco, and aspirin. For the Fox Insight cohort, information on the Environmental Exposure Questionnaires for coffee, tobacco, and aspirin, which are also based on the PD-RFQs, were provided through one-time questionnaires that are part of the online clinical assessment^50^. In this study group, patients were classified as coffee consumers if they regularly drank caffeinated coffee at least once per week over a period of at least 6 months. Patients were classified as tobacco users if they have ever used tobacco, or when available, if they smoked more than 100 cigarettes in their lifetime or if they smoked at least one cigarette per day over a minimum period of 6 months or if they used smokeless tobacco at least once per day for more than 6 months. Lastly, patients were classified as aspirin users if they took at least two pills per week over a minimum of 6 months. No distinctions were made between current and former users of these lifestyle factors.

Duration of caffeine consumption, smoking, and aspirin intake were estimated according to the age the patients started using either substance subtracted from the age at termination. If the patients terminated the consumption after their AAO, the age the patients started was subtracted from their AAO. Periods, where the patients stopped regularly consuming, were subtracted from the overall duration.

Coffee drinking dosage was defined as the average number of cups of coffee per week the patients drank within the drinking duration time. Smoking dosage was estimated as cigarettes smoked per day within the smoking duration time. Aspirin dosage was defined as pills per week the patients took within the aspirin intake duration time. The number of cups of coffee for non-drinkers, cigarettes for non-smokers, and pills per week for aspirin non-users was set to zero. In addition, coffee drinking duration for non-drinkers, smoking duration for non-smokers, and aspirin intake duration for aspirin non-users was set to zero.

### Statistical analysis

Statistical analyses were performed using R v4.3.0^56^. Multiple linear regression models were used to evaluate the association between AAO, PGS, and lifestyle factors in patients with PD. All linear regression models were validated by evaluating diagnostic plots (Residuals vs. Fitted, Q–Q Residuals, Scale-Location, and Residuals vs. Leverage) and outliers were removed if applicable. In the linear models, AAO was used as the dependent variable and the PGS and/or the lifestyle factors as the independent variables. Estimates (*β*), standard errors (*SE*) and *p* values were reported. To adjust for potential confounders, sex and the first two principal components (PCs) were included as covariables in the models. Reported *p* values were not corrected for multiple testing because they did not follow an “a priori” hypothesis and results were exploratory. Lifestyle factors were handled in three different ways in the regression models: (1) binary (ever–never/yes–no indication), (2) dosage as a continuous variable, and (3) duration as a continuous variable. In a second set of regression models, data from patients with PD and healthy controls was used to estimate Cox proportional hazards models. Here we modeled AAO from the cumulative number of lifestyle factors used (R package *survival*) and we used AAO for patients with PD and AAE with censoring for healthy controls. The total number of the three lifestyle factors coffee, tobacco, and aspirin the participants used were included as the numbers zero to three. The sex and the study site were additionally included as covariables since genetic data and thus genetic PCs were not available for all participants. Survival plots and forest plots were generated to visualize the Cox proportional hazards model using the *ggsurvplot* (R package *survminer*) and *forest_model* function (R package *forestmodel*). Regression coefficients, hazards ratios (*HR*), 95% confidence intervals (95% CI) and *p* values were reported. To compare the model accuracies of different linear regression models, the adjusted deviance-based *R*^2^ was calculated using the *adjR2* function (R package *glmtoolbox*). To compare the AAO ranges in patients with PD with respect to their PGS, the patients were stratified into quartiles according to their PGS and the difference in AAO between groups was calculated. In addition, to compare the effect sizes of PGS and lifestyle factors on AAO, the PGS was categorized into “low PGS” and “high PGS” according to the median PGS and participants were stratified into the subgroups that either used no protective lifestyle factor or that used all three lifestyle factors.

### Ethics approval

Approval was obtained from the Ethics Committee of the University of Lübeck. We confirm that all analyses were performed in accordance with relevant guidelines and regulations.

### Consent to participate

Informed consent was obtained from all individual participants included in the study.

## Results

### Relationship between PGS and AAO

First, to validate the PD-specific PGS in this study group, the PGS values of patients with PD and healthy controls were assessed. In a case–control comparison, the AUC for the ROC curves of the standardized PGS was 0.67, which was comparable to the AUC obtained in the original study^2^.

To analyze the association between the PGS and AAO in patients with PD, a linear regression model including sex and the first two PCs as covariates was used. The PGS showed a negative association with AAO (*β* = − 1.07, SE = 0.21, *p* = 6 × 10^–7^). Thus, if the PGS is increased by one standard deviation (*SD*), the estimated AAO is approximately one year earlier in patients with PD.

We also assessed the AAO ranges in patients with PD by stratifying and comparing the first and last PGS quartiles. Patients with PD in the first PGS quartile had a median AAO of 63 years (range: 31–85 years), while PD patients in the last PGS quartile had a median AAO of 61 years (range: 34–83 years), showing a difference in the median AAO of 2 years in these two groups.

### Relationship between lifestyle factors and AAO

We replicated our previous findings from the Fox Insight cohort^32^ in the AMP-PD/PPMI cohort. In the linear regression model, coffee drinking duration was positively associated with AAO (*β* = 0.19, SE = 0.04, *p* = 3 × 10^–5^). In addition, tobacco use showed a positive association with AAO (*β* = 3.21, SE = 0.50, *p* = 2 × 10^–10^). We also observed positive associations between aspirin use (*β* = 7.35, SE = 1.50, *p* = 3 × 10^–6^), aspirin dosage (*β* = 0.88, SE = 0.21, *p* = 7 × 10^–5^), and aspirin duration (*β* = 0.55, SE = 0.17, *p* = 0.0013) and the AAO. To investigate the additive effect of the three lifestyle factors, we coded them by the cumulative number of factors the patients consumed. In this linear regression model, the use of three (*β* = 6.34, SE = 2.86, *p* = 0.0284) protective lifestyle factors showed an association with AAO, while the use of one lifestyle factor (*β* = 0.21, SE = 2.37, *p* = 0.9284) or two lifestyle factors (*β* = 4.30, SE = 2.46, *p* = 0.0827) was not associated with AAO. Interestingly, when including all three factors separately in the same model to predict AAO, aspirin was still associated with AAO (*β* = 7.40, SE = 1.54, *p* = 4 × 10^–6^) and the other associations diminished. Although all three protective factors are associated with AAO, aspirin is shown to be a better predictor of AAO when only one lifestyle factor was included in the model (*R*^2^ = 0.1694; aspirin (yes/no), sex, PC1, and PC2 in the model) compared to coffee and tobacco use (*R*^2^ = 0.0207, *R*^2^ = 0.0287; coffee (yes/no) or tobacco (ever/never), sex, PC1, and PC2 in the model).

**Figure 1:**
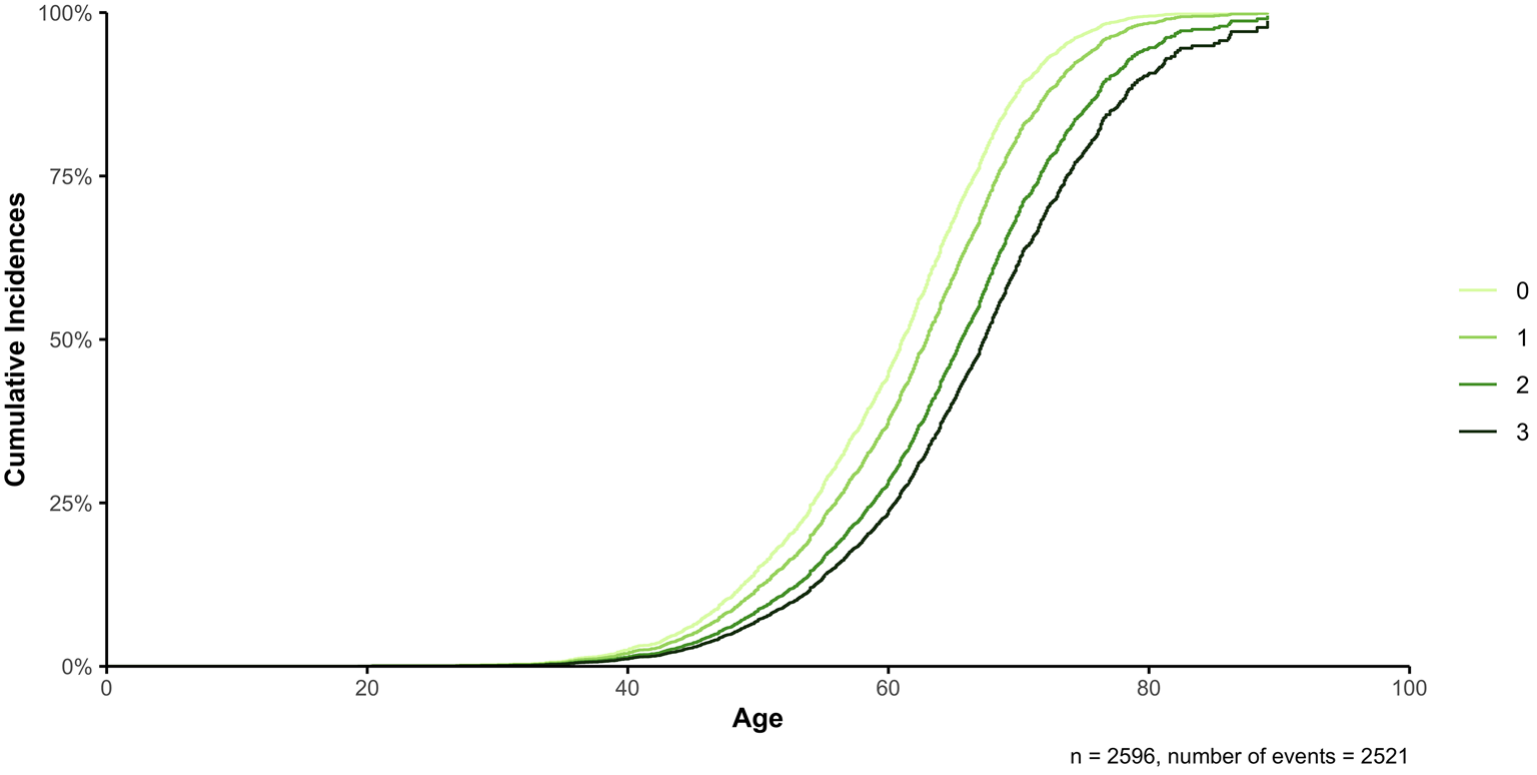
Additive effects of the lifestyle factors coffee drinking, tobacco use, and aspirin intake on the AAO of PD patients, while censoring with the AAE of healthy controls. The different curves describe the cumulative number (0–3) of protective lifestyle factors (coffee drinking, tobacco use, and aspirin intake) the participants used. A Cox proportional hazards model was used to investigate the difference in AAO with respect to the number of protective lifestyle factors used, while censoring with the AAE of healthy controls. The sex and study site were additionally included as covariates (→ coxph(formula = Surv(AAO/AAE, Diagnosis) ~ Coffee/Tobacco/Aspirin + Sex + Study, data = data)).

In a combined analysis of individuals from both AMP-PD/PPMI and Fox Insight using Cox proportional hazards models on the AAO of patients with PD while including the AAE of healthy controls, we first included the lifestyle factors as separate factors. Coffee (HR = 0.75, 95% CI 0.68–0.82, *p* = 1 × 10^–9^), tobacco (HR = 0.78, 95% CI 0.72–0.85, *p* = 8 × 10^–9^), and aspirin (HR = 0.66, 95% CI 0.60–0.71, *p* < 2 × 10^–16^) showed a reduction in the hazard ratio compared to no use by 25%, 22%, and 34%, respectively (Supplementary Fig. 1). In addition, we assessed the dosage and duration for each lifestyle factors in Cox proportional hazards models as a continuous variable, again showing a reduction in the hazard ratio for coffee (dosage: HR = 0.99, 95% CI 0.99–1.00, *p* = 0.0006, duration: HR = 0.99, 95% CI 0.98–0.99, *p* < 2 × 10^–16^), tobacco (dosage: HR = 0.99, 95% CI 0.99–1.00, *p* = 8 × 10^–6^, duration: HR = 0.99, 95% CI 0.98–0.99, *p* = 9 × 10^–8^), and aspirin (dosage: HR = 0.97, 95% CI 0.96–0.98, *p* = 6 × 10^–11^, duration: HR = 0.97, 95% CI 0.97–0.98, *p* = 1 × 10^–14^). To investigate the potential additive effect between all three lifestyle factors, they were coded by the cumulative number of lifestyle factors the participants consumed as above. In the Cox proportional hazards model, the use of one (HR = 0.79, 95% CI 0.70–0.89, *p* = 0.0001), two (HR = 0.56, 95% CI 0.49–0.63, *p* < 2 × 10^–16^), or three (HR = 0.45, 95% CI 0.39–0.53, *p* < 2 × 10^–16^) of the selected lifestyle factors showed a reduction in the hazard ratio compared to the use of none of these lifestyle factors by 21%, 44%, and 55%, respectively, indicating a later AAO when using more lifestyle factors (Table 2, Fig. 1, Supplementary Fig. 2).

**Table 2:** Cox proportional hazards model to investigate the additive effects between the use of the lifestyle factors coffee drinking, tobacco use, and aspirin intake (cumulative number (0–3)) on the AAO of PD, while censoring with the AAE of healthy controls.

	Regression coefficient	Hazard ratio (95% CI)	*p* value
*n* = 2596, *n* events = 2521			
Coffee/Tobacco/Aspirin (1)	− 0.2362	0.7896 (0.6992, 0.8918)	0.0001*
Coffee/Tobacco/Aspirin (2)	− 0.5855	0.5568 (0.4918, 0.6305)	** < 2 × 10**^–16^*
Coffee/Tobacco/Aspirin (3)	− 0.7925	0.4527 (0.3851, 0.5321)	** < 2 × 10**^–16^*
Sex (female)	0.1168	1.1239 (1.0366, 1.2186)	0.0046*
Study (Fox Insight)	0.3097	1.3631 (1.1482, 1.6181)	0.0004*

coxph (formula = Surv(AAO/AAE, Diagnosis) ~ Coffee/Tobacco/Aspirin + Sex + Study, data = data).

Baseline categories: Coffee/Tobacco/Aspirin = 0, Sex = male, Study = AMP-PD/PPMI.

*AAO* age at onset; *AAE* age at examination; *PD* Parkinson’s disease; *CI* confidence interval.

*p value < 0.05 are highlighted in bold.

We assessed the AAO ranges in the different groups of lifestyle factor exposures. In the subgroup of patients with PD that used no protective lifestyle factor, the median AAO was 57 years (range: 19–78 years), while patients with PD that drank coffee and used tobacco and aspirin had a median AAO of 66 years (range: 38–86 years), indicating a difference in the median AAO of 9 years in these two groups.

### Additive and interaction effects between PGS and lifestyle factors on AAO

Next, we explored the additive and interactive effects of the PGS and lifestyle factors on AAO in linear regression models. In the additive models including the lifestyle factor coffee drinking, the PGS was not associated with AAO when coffee drinking (binary, dosage, duration) was included as a covariate (Table 3). However, the positive association between the coffee drinking duration and AAO was still robust (*β* = 0.18, SE = 0.04, *p* = 5 × 10^–5^). Further, there were no interactions between the PGS and coffee drinking in the patients with PD.

**Table 3:** Linear model on the association of PGS and coffee drinking with AAO in the PD study group.

	Estimate	Standard error	*p* value
Coffee drinking (binary) (*n* = 139)^1^			
Intercept	56.9608	1.9194	** < 2 × 10**^–16^*
PGS	− 1.3514	0.6955	0.0541
Coffee drinking (binary)	1.1929	1.8383	0.5175
Sex (male)	3.4321	1.6566	0.0402*
PC1	− 8.3114	71.1118	0.9071
PC2	− 28.5586	56.6419	0.6150
Coffee drinking dosage (*n* = 139)^1^			
Intercept	58.1752	1.6649	** < 2 × 10**^–16^*
PGS	− 1.2006	0.7056	0.0912
Coffee drinking dosage	− 0.0414	0.0816	0.6125
Sex (male)	3.7245	1.6396	0.0247*
PC1	− 56.0175	67.0364	0.4049
PC2	− 37.4508	57.1142	0.5131
Coffee drinking duration (*n* = 100)^1^			
Intercept	55.3345	1.8344	** < 2 × 10**^–16^*
PGS	− 1.0068	0.8288	0.2275
Coffee drinking duration	0.1840	0.0432	**5 × 10**^–5^*
Sex (Male)	− 1.1479	1.8432	0.5349
PC1	101.8483	84.7233	0.2323
PC2	− 79.4870	60.2628	0.1904
Coffee drinking (binary) (*n* = 138)^2^			
Intercept	57.3700	2.0912	** < 2 × 10**^–16^*
PGS	− 2.3622	1.4141	0.0972
Coffee drinking (binary)	0.9643	2.2095	0.6632
Sex (male)	3.1499	1.6543	0.0591
PC1	− 13.5675	70.8490	0.8484
PC2	− 9.0768	57.0590	0.8739
PGS:Coffee drinking (binary)	1.0425	1.6328	0.5243
Coffee drinking dosage (*n* = 139)^2^			
Intercept	58.5453	1.7536	** < 2 × 10**^–16^*
PGS	− 1.7430	1.0617	0.1030
Coffee drinking dosage	− 0.0759	0.0960	0.4305
Sex (male)	3.6559	1.6459	0.0280*
PC1	− 53.6120	67.2625	0.4269
PC2	− 36.9398	57.2335	0.5198
PGS:Coffee drinking dosage	0.0595	0.0869	0.4948
Coffee drinking duration (*n* = 100)^2^			
Intercept	55.4035	1.9016	** < 2 × 10**^–16^*
PGS	− 1.1151	1.1070	0.3164
Coffee drinking duration	0.1797	0.0522	0.0009*
Sex (male)	− 1.1331	1.8555	0.5429
PC1	103.6788	86.0536	0.2313
PC2	− 79.7335	60.6014	0.1915
PGS:Coffee drinking duration	0.0062	0.0419	0.8822

*PGS* polygenic score; *AAO* age at onset; *PD* Parkinson’s disease; *PC* principal component; *glm* generalized linear model.

*p value < 0.05 are highlighted in bold.

^1^glm(formula = AAO ~ PGS + Coffee drinking + Sex + PC1 + PC2, family = gaussian, data = data).

^2^glm(formula = AAO ~ PGS * Coffee drinking + Sex + PC1 + PC2, family = gaussian, data = data).

When we investigated the association between PGS and tobacco use in an additive regression model, both the PGS (*β* = − 1.11, SE = 0.24, *p* = 4 × 10^–6^) and tobacco use (binary ever–never indication) (*β* = 3.16, SE = 0.50, *p* = 3 × 10^–10^) showed associations with AAO (Table 4). However, when dosage of tobacco was included, both the association between tobacco dosage and AAO (*β* = 0.11, SE = 0.08, *p* = 0.2023) as well as between PGS and AAO (*β* = − 1.31, SE = 0.70, *p* = 0.0629) diminished. Similarly, no association between the duration of tobacco use and AAO was found (*β* = 0.17, SE = 0.10, *p* = 0.0910), while the association between the PGS and AAO (*β* = − 1.44, SE = 0.73 *p* = 0.0496) was still robust. In addition, there were no interactions between the PGS and tobacco use with AAO.

**Table 4:** Linear model on the association of PGS and tobacco use with AAO in the PD study group.

	Estimate	Standard error	*p* value
Tobacco use (binary) (*n* = 1650)^1^			
Intercept	60.3997	0.4749	** < 2 × 10**^–16^*
PGS	− 1.1107	0.2398	**4 × 10**^–6^*
Tobacco use (binary)	3.1643	0.5006	**3 × 10**^–10^*
Sex (Male)	− 0.3431	0.5089	0.5002
PC1	− 72.7674	18.6688	0.0001*
PC2	− 4.5250	17.0231	0.7904
Tobacco use dosage (*n* = 136)^1^			
Intercept	57.6722	1.5276	** < 2 × 10**^–16^*
PGS	− 1.3125	0.6996	0.0629
Tobacco use dosage	0.1061	0.0828	0.2023
Sex (Male)	3.4475	1.6796	0.0421*
PC1	− 46.5124	67.3827	0.4913
PC2	− 35.0421	58.9671	0.5534
Tobacco use duration (*n* = 126)^1^			
Intercept	57.4759	1.5461	** < 2 × 10**^–16^*
PGS	− 1.4445	0.7282	0.0496*
Tobacco use duration	0.1686	0.0989	0.0910
Sex (male)	3.7752	1.7293	0.0310*
PC1	− 30.4355	69.4755	0.6621
PC2	− 49.0272	61.5275	0.4271
Tobacco use (binary) (*n* = 1650)^2^			
Intercept	60.3051	0.4916	** < 2 × 10**^–16^*
PGS	− 0.9605	0.3132	0.0022*
Tobacco use (binary)	3.4028	0.5942	**1 × 10**^–8^*
Sex (male)	− 0.3596	0.5094	0.4804
PC1	− 72.5440	18.6737	0.0001*
PC2	− 4.7758	17.0287	0.7792
PGS:Tobacco use (binary)	− 0.3599	0.4829	0.4561
Tobacco use dosage (*n* = 136)^2^			
Intercept	57.7039	1.5443	** < 2 × 10**^–16^*
PGS	− 1.3800	0.8034	0.0883
Tobacco use dosage	0.0969	0.0985	0.3272
Sex (male)	3.4725	1.6921	0.0422*
PC1	− 46.2088	67.6583	0.4959
PC2	− 33.8824	59.5670	0.5705
PGS:Tobacco use dosage	0.0123	0.0714	0.8630
Tobacco use duration (*n* = 126)^2^			
Intercept	57.4071	1.5621	** < 2 × 10**^–16^*
PGS	− 1.3076	0.8139	0.1108
Tobacco use duration	0.1977	0.1251	0.1169
Sex (male)	3.7388	1.7381	0.0335*
PC1	− 31.6402	69.7955	0.6511
PC2	− 52.7653	62.5193	0.4004
PGS:Tobacco use duration	− 0.0324	0.0848	0.7033

*PGS* polygenic score; *AAO* age at onset; *PD* Parkinson’s disease; *PC* principal component; glm, generalized linear model.

*p value < 0.05 are highlighted in bold.

^1^glm(formula = AAO ~ PGS + Tobacco use + Sex + PC1 + PC2, family = gaussian, data = data).

^2^glm(formula = AAO ~ PGS * Tobacco use + Sex + PC1 + PC2, family = gaussian, data = data).

We further explored the association between the PGS and aspirin intake on AAO in an additive regression model. When aspirin intake was included as a binary yes–no indication, both the PGS (*β* = − 1.58, SE = 0.64, *p* = 0.0149) and aspirin intake (*β* = 7.62, SE = 1.48, *p* = 9 × 10^–7^) were associated with AAO in patients with PD (Table 5). Similarly, when the aspirin intake dosage was included in the model, the PGS (*β* = − 1.57, SE = 0.70, *p* = 0.0260), as well as aspirin intake dosage (*β* = 0.88, SE = 0.21, *p* = 5 × 10^–5^), were associated with AAO. However, when including the aspirin intake duration in the model, aspirin showed an association with AAO (*β* = 0.56, SE = 0.16, *p* = 0.0009), while the association between PGS and AAO diminished (*β* = − 1.35, SE = 0.71, *p* = 0.0602). There was further no interaction between PGS and aspirin intake in all interaction models.

**Table 5:** Linear model on the association of PGS and aspirin intake with AAO in the PD study group.

	Estimate	Standard error	*p* value
Aspirin intake (binary) (*n* = 140)^1^			
Intercept	55.7965	1.4149	** < 2 × 10**^–16^*
PGS	− 1.5838	0.6418	0.0149*
Aspirin intake (binary)	7.6159	1.4780	**9 × 10**^–7^*
Sex (male)	2.4371	1.5181	0.1108
PC1	− 22.9003	61.8372	0.7117
PC2	− 36.6181	52.0627	0.4831
Aspirin intake dosage (*n* = 134)^1^			
Intercept	56.5418	1.4797	** < 2 × 10**^–16^*
PGS	− 1.5678	0.6959	0.0260*
Aspirin intake dosage	0.8817	0.2106	**5 × 10**^–5^*
Sex (Male)	2.1982	1.6181	0.1767
PC1	− 34.1742	64.5357	0.5974
PC2	− 18.0128	54.6272	0.7421
Aspirin intake duration (*n* = 115)^1^			
Intercept	55.2310	1.5322	** < 2 × 10**^–16^*
PGS	− 1.3545	0.7133	0.0602
Aspirin intake duration	0.5609	0.1649	0.0009*
Sex (male)	3.6247	1.7247	0.0379*
PC1	43.9351	72.9233	0.5481
PC2	26.9794	58.3585	0.6448
Aspirin intake (binary) (*n* = 140)^2^			
Intercept	55.7961	1.4853	** < 2 × 10**^–16^*
PGS	− 1.5833	0.8587	0.0674
Aspirin intake (binary)	7.6168	1.7721	**3 × 10**^–5^*
Sex (male)	2.4371	1.5249	0.1124
PC1	− 22.8956	62.3001	0.7138
PC2	− 36.6181	52.2582	0.4847
PGS:Aspirin intake (binary)	− 0.0012	1.2985	0.9993
Aspirin intake dosage (n = 134)^2^			
Intercept	56.7069	1.5477	** < 2 × 10**^–16^*
PGS	− 1.7694	0.8787	0.0462*
Aspirin intake dosage	0.8399	0.2386	0.0006*
Sex (male)	2.1354	1.6320	0.1931
PC1	− 35.7510	64.8871	0.5826
PC2	− 19.4946	54.9510	0.7234
PGS:Aspirin intake dosage	0.0738	0.1952	0.7061
Aspirin intake duration (*n* = 115)^2^			
Intercept	55.3354	1.5472	** < 2 × 10**^–16^*
PGS	− 1.5324	0.7778	0.0514
Aspirin intake duration	0.5162	0.1823	0.0055*
Sex (Male)	3.5763	1.7319	0.0413*
PC1	47.1666	73.3544	0.5216
PC2	25.1460	58.6203	0.6688
PGS:Aspirin intake duration	0.0809	0.1387	0.5609

*PGS* polygenic score; *AAO* age at onset; *PD* Parkinson’s disease; *PC* principal component; glm, generalized linear model.

*p value < 0.05 are highlighted in bold.

^1^glm(formula = AAO ~ PGS + Aspirin intake + Sex + PC1 + PC2, family = gaussian, data = data).

^2^glm(formula = AAO ~ PGS * Aspirin intake + Sex + PC1 + PC2, family = gaussian, data = data).

### Impact of PGS and lifestyle factors on AAO

Since the association between PGS and AAO diminished in some models including lifestyle factors as covariables, we investigated the impact PGS and lifestyle factors have on the AAO in PD. In a first approach to compare the effect sizes of PGS and lifestyle factors on AAO, we categorized the PGS into “low PGS” and “high PGS” according to the median PGS and stratified participants into the subgroups that either used no protective lifestyle factor or that used all three lifestyle factors. In the subgroup of participants that used no protective lifestyle factor, a high PGS showed a 3.03 times higher expected hazard of PD as compared to a low PGS (HR = 3.03, 95% CI 1.05–8.78, *p* = 0.0409). In contrast, in the subgroup of participants that used all three lifestyle factors, there was no increased hazard ratio (HR = 1.21, 95% CI 0.49–2.99, *p* = 0.6863) (Supplementary Fig. 3).

We further investigated the model goodness-of-fit of the linear models using the adjusted deviance-based *R*^2^. The model assessing the association between PGS and AAO, while using sex and the first two PCs as covariables, had an adjusted *R*^2^ of 0.0141. In contrast, the linear model evaluating the association between the three lifestyle factors and AAO with the same covariables had an adjusted *R*^2^ of 0.0856, when the lifestyle factors were coded as cumulative quantitative numbers. In the combined linear model, determining the additive association between PGS and the three lifestyle factors with the same covariables as before showed an adjusted *R*^2^ of 0.1039.

### Relationship between lifestyle factors and AAO in *GBA1*-PD

To investigate if the individual and combined effects of the lifestyle factors coffee, tobacco, and aspirin are exclusive to idiopathic PD or if these effects can also be found in patients who carry *GBA1* variants, which are considered some of the strongest genetic risk variants for PD, we examined the relationship between the protective lifestyle factors and AAO in an additional study group of patients with *GBA1*-PD from Fox Insight. In the linear regression model, coffee drinking duration was positively associated with AAO (*β* = 0.24, SE = 0.05, *p* = 7 × 10^–7^) in *GBA1*-PD (Supplementary Table 1). In addition, we observed a positive association between tobacco use and AAO (*β* = 3.65, SE = 1.58, *p* = 0.0223) and between aspirin intake duration and AAO (*β* = 0.48, SE = 0.21, *p* = 0.0224). When including all three lifestyle factors separately in the same model to predict AAO, only tobacco use was associated with AAO (*β* = 6.78, SE = 2.17, *p* = 0.0024), which contrasts with the results found in idiopathic PD. To examine the combined effect of the three lifestyle factors in more detail, we used as influence variable the cumulative number of factors in the Cox proportional hazards model. We observed a protective trend for this variable. With no lifestyle factor as reference, we observed an *HR* of 0.85 for the use of one (95% CI 0.40–1.78, *p* = 0.6648), *HR* of 0.47 for the use of two (95% CI 0.23–0.97, *p* = 0.0410), and an *HR* of 0.35 for the use of three lifestyle factors (95% CI 0.14–0.86, *p* = 0.0216). Of note, the use of one lifestyle factor did not show a significant reduction in the hazard ratio compared to the use of none of these lifestyle factors, which could be a problem of statistical power. However, the use of two or three lifestyle factors showed a significant reduction in the hazard ratio by 53% and 65%, indicating a protective effect on the AAO when using more lifestyle factors.

In an approach to directly compare the relationship of lifestyle factors on AAO between patients with *GBA1*-PD and PD patients without known mutations, we performed the linear regression models including all patients with PD and using the *GBA1* mutation carrier status as another covariate. In the linear regression models, all lifestyle factors showed a positive association with AAO (Supplementary Table 2). Interestingly, an association between *GBA1* mutation carrier status and AAO was further found in the models with coffee drinking dosage (*β* = 2.10, SE = 0.90, *p* = 0.0197), tobacco use (binary) (*β* = 1.74, SE = 0.79, *p* = 0.0280), and tobacco use duration (*β* = 1.84, SE = 0.88, *p* = 0.0357). Therefore, *GBA1* mutation carrier status did not affect the impact of these environmental factors on AAO.

## Discussion

In this study, we have investigated the association between the PGS, calculated based on a previously proposed composition of 1805 variants^2^, and the AAO in patients with PD and determined the interaction between the PGS and the lifestyle factors coffee drinking, tobacco use, and aspirin intake on the AAO in PD.

We found that the PGS not only allows discrimination between PD cases and controls^2,4^ but also showed a negative correlation with AAO^4,11,13^, indicating that an increase of the PGS by one SD leads to an approximately one year earlier AAO in patients with PD. This relationship between PGS and AAO was also robust when adjusting for potentially confounding covariables (i.e., sex and ancestry as represented by the first two principal components). These results demonstrate that the genetic composition, represented by the PGS, adds to understanding the variance in AAO in patients with PD. However, with a range in AAO of 70 years in this study group, more influencing factors and cofounders need to be considered.

The protective effect of environmental and lifestyle factors that decrease the risk of developing PD, influence initial PD-related symptoms and progression, and delay AAO has already been known for years^57,58^. However, how these lifestyle factors interact and which combined effect they have on PD AAO remains unresolved. Our group has previously presented a protective effect of coffee, tobacco, and aspirin on the AAO of patients with PD from the Fox Insight study^32^, which we further replicated in the AMP-PD/PPMI study group here. This correlation between lifestyle factors and PD AAO consistently highlights the importance of investigating this interplay further. In a more detailed analysis of the combined effect of the three protective lifestyle factors coffee, tobacco, and aspirin on AAO, we found that the use of either one, two, or three lifestyle factors led to a reduction in the hazard ratio by 21%, 44%, and 55%, respectively, in comparison to no use. As later AAO will tend to be positively associated with lifestyle factor use due to longer observation time, these models were censored with controls to account for this bias. Nevertheless, it is important to take into account that the baseline hazards are already biased as the number of cases and controls are not population representative, which leads to a hazard overestimation for cases and an underestimation for controls. These hazard ratio values are consistent with additive, i.e., independent effects on the logit scale of the lifestyle factors with no synergistic interaction, indicating different underlying mechanisms that lead to the later AAO. Deciphering these mechanisms of action is important to develop suitable therapeutic strategies to delay the AAO of patients with idiopathic PD. In addition, by separating former and current lifestyle factor users, possible long-lasting effects could be predicted. Interestingly, aspirin seems to have a larger effect on AAO than coffee or tobacco. The effect of aspirin intake on PD risk is still disputed and the prevalence of NSAIDs use appears to be comparable in the general population and patients with PD^59,60^. The prevalence of NSAID intake in the general population varies depending on the definition of NSAID usage, which may include restrictions by categories such as prescription and non-prescription drugs, NSAID doses per pill, and reason for use (e.g., pain (e.g. after a surgery, back pain, headache, menstrual pain), prevention of cardiovascular diseases, or for antipyretic use). In the US general population, the prevalence of aspirin intake, which is the most frequently prescribed NSAID^61^, has been reported to be approximately 50% in adults^62,63^. Therefore, the prevalence of aspirin intake in the general population, including the PD population, is high, although not as high as for coffee drinking. Given that inflammation is a crucial pathophysiological pathway in PD^64^, the anti-inflammatory effect of aspirin might have a protective impact on PD AAO. Although a positive effect of non-steroidal anti-inflammatory drugs on PD risk is contentious^22,59,60^, it is well-known that sustained neuroinflammation leads to the progressive degeneration of dopaminergic neurons^65^. The intake of anti-inflammatory drugs such as aspirin in the prodromal phase, when neuronal degeneration has already started, might therefore slow this process resulting in a later AAO. In addition, studying peripheral immune system alterations and their interactions with aspirin intake and AAO might be beneficial to decipher the underlying mechanisms. The relationship between vascular disorders and PD is controversial, however, it could have played a role in the inter-relationship of aspirin intake, tobacco, and PD. In our cohort after adjusting for comorbidities such as lung diseases, heart diseases, arthritis, back pain, and surgeries with anesthesia as covariates our results remain robust^32^. We were limited to the available data on comorbidities in these patients and could not extensively look into the type of vascular disease (i.e., myocardial infarction or stroke). In contrast, the protective effect of aspirin on the AAO of patients with *GBA1*-PD was not as pronounced, indicating that the neuroinflammatory mechanisms leading to neurodegeneration might diverge from patients with idiopathic PD or be masked by the genetic susceptibility in *GBA1*-PD. However, as sample sizes for patients with *GBA1*-PD were small (*n* = 159), findings need to be interpreted with caution. Assuming a significance level of 0.05 the power of the coffee assessment is 0.39, of the tobacco assessment it is 0.6, and of the aspirin assessment it is 0.34 for the available patients with *GBA1*-PD. To increase the statistical power to 0.8 with a constant effect size, *n* = 188 coffee drinkers and *n* = 27 non-coffee drinkers, *n* = 82 tobacco users and *n* = 144 non-tobacco users, and *n* = 88 aspirin users and *n* = 200 non-aspirin users would be needed. In contrast, for patients with idiopathic PD the power in all three assessments is almost 1. Although the pro-inflammatory signaling does not seem to be related to the PD subtype and there is no evidence of a difference in the immune response between idiopathic PD, monogenic forms of PD (e.g., *LRRK2*-PD), and strong risk factor carriers such as *GBA1*-PD^66^, those PD subtypes present with different phenotypes and might need to be treated differently. Thus far, it is not clear how the underlying mechanisms work and if they differ in the different subtypes of PD. However, we have already seen that the effects of environmental and lifestyle factors as well as specific genetic risk factors on AAO vary in different subtypes of PD, especially between monogenic forms of PD and idiopathic PD^67^. To follow up on this, future larger-scale studies including patients with monogenic forms of PD or who carry strong risk factors (e.g., *LRRK2*-PD, *GBA1*-PD, or *PRKN/PINK1*-PD), are important to target the effect of anti-inflammatory lifestyle factors on PD AAO. Especially patients with *PRKN/PINK1*-related PD could be of particular interest as an early AAO is a clinical hallmark of these patients.

In order to investigate the additive and interaction effects of the lifestyle factors together with the PGS on AAO, we applied linear models, showing additive and independent effects of PGS and tobacco use on AAO as well as of PGS and aspirin intake on AAO, with opposite directionality of the PGS and the lifestyle factors. The possibility of an interaction between PGS and lifestyle factors cannot be entirely ruled out. In fact, an interaction between PGS and smoking was reported in two recent studies^6,7^. In our study, we found a three times higher expected hazard of PD in the subgroup of participants that used no protective lifestyle factor and had a high PGS as compared to patients with a low PGS. However, the results are thus far only preliminary and need to be interpreted with caution. Nevertheless, they indicate that the PGS is more important for persons that use no protective lifestyle factors. We also found the three lifestyle factors to explain the AAO in patients with PD more accurately than the PGS (Lifestyle factor model: *R*^2^ = 0.0856, PGS model: *R*^2^ = 0.0141).

Although there are known gene-environment interactions of coffee and tobacco with PD^33–36^, none of the variants included in the calculation of the PGS are located within genes known to show interactions with coffee, smoking, or aspirin. Since this PGS is based on common variants associated with PD risk, it is pathway-independent and different mechanisms can lead to the earlier AAO when having a high PGS or the delayed AAO when using protective lifestyle factors. In contrast, pathway-dependent PGSs such as the mitochondrial polygenic score^67,68^ have been shown to interact with lifestyle factors such as pesticides or caffeinated beverages.

Limitations of our study include clinical and genetic data harmonization. The use of data from different cohorts poses the problem of overcoming potential inconsistencies due to differences in the way of data collection. To help overcome this problem, we corrected for the study site in our Cox proportional hazards models including lifestyle data from AMP-PD/PPMI and Fox Insight and also corrected for the first two principal components in all genetic data analyses to account for genetic differences due to ethnic diversity or differences in the type of genetic data collection. Another limitation was that the clinical data provided by the three cohorts sparsely overlapped with genetic data, resulting in small sample sizes in some of the subgroups. Nevertheless, we showed that lifestyle factors have an important effect on PD AAO that is even greater than that of a combined genetic risk. In future studies, this analysis needs to be replicated in a larger study group with diverse ancestral backgrounds. Since the GWAS that was used for the PGS calculation was performed in a European ancestry population, we only included PD patients and controls with European ancestry in our study group. The lack of ancestry and ethnic diversity in large-scale genetic studies is a well-known problem^69–71^. To completely unravel the genetic mechanisms that lead to developing PD, future studies must be inclusive of patients from all cultural and genetic backgrounds.

In conclusion, this study is the first to assess the combined effect of the PD-specific PGS together with coffee drinking, tobacco use, and aspirin intake on the AAO of patients with PD and adds to understanding this complex disease. Our results further indicate a potential neuroprotective role of the anti-inflammatory drug aspirin resulting in a later AAO in PD. Aspirin might play an important protective part in the inflammatory processes that could lead to neurodegeneration in PD. Thus far, these results are only exploratory, because they did not follow an “a priori” hypothesis and the results of the survival analyses only apply to the investigated study group. Therefore, further validation is essential. Nevertheless, our findings underline the importance of investigating both genetic disposition and external influences such as environmental and lifestyle factors to unravel the likelihood of disease manifestation and the variable phenotype presented in patients with PD.

## Supplementary Information

Supplementary Information.

## Data Availability

Data used in the preparation of this article were obtained from the Accelerating Medicine Partnership® (AMP®) Parkinson’s Disease (AMP PD) Knowledge Platform. For up-to-date information on the study, visit https://www.amp-pd.org. The data that support the findings of this study are available from the Accelerating Medicine Partnership® (AMP®) Parkinson’s Disease Knowledge Platform, but restrictions apply to the availability of these data, which were used under license for the current study, and so are not publicly available. Data are however available from the authors upon reasonable request (joanne.trinh@neuro.uni-luebeck.de) and with permission of the Accelerating Medicine Partnership® (AMP®) Parkinson’s Disease Knowledge Platform. Data used in the preparation of this manuscript were obtained from the Fox Insight database (https://foxinsight-info.michaeljfox.org/insight/explore/insight.jsp) on 22/05/2023. For up-to-date information on the study, visit https://foxinsight-info.michaeljfox.org/insight/explore/insight.jsp. Data used in the preparation of this article were obtained from the Parkinson’s Progression Markers Initiative (PPMI) database (www.ppmi-info.org/access-data-specimens/download-data), RRID:SCR_006431. For up-to-date information on the study, visit www.ppmi-info.org.

## References

[1] Jia, F., Fellner, A. & Kumar, K. R. Monogenic Parkinson’s disease: Genotype, phenotype, pathophysiology, and genetic testing. *Genes (Basel)*10.3390/genes13030471 (2022). 35328025 10.3390/genes13030471 PMC8950888

[2] Nalls, M. A. *et al.* Identification of novel risk loci, causal insights, and heritable risk for Parkinson’s disease: A meta-analysis of genome-wide association studies. *Lancet Neurol.* **18**, 1091–1102. 10.1016/S1474-4422(19)30320-5 (2019). 31701892 10.1016/S1474-4422(19)30320-5 PMC8422160

[3] Chang, D. *et al.* A meta-analysis of genome-wide association studies identifies 17 new Parkinson’s disease risk loci. *Nat. Genet.* **49**, 1511–1516. 10.1038/ng.3955 (2017). 28892059 10.1038/ng.3955 PMC5812477

[4] Koch, S. *et al.* Validity and prognostic value of a polygenic risk score for Parkinson’s disease. *Genes (Basel)*10.3390/genes12121859 (2021). 34946808 10.3390/genes12121859 PMC8700849

[5] Li, W. W. *et al.* Association of the Polygenic risk score with the incidence risk of Parkinson’s disease and cerebrospinal fluid alpha-synuclein in a Chinese cohort. *Neurotox. Res.* **36**, 515–522. 10.1007/s12640-019-00066-2 (2019). 31209785 10.1007/s12640-019-00066-2

[6] Reynoso, A. *et al.* Gene-environment interactions for Parkinson’s disease. *Ann. Neurol.*10.1002/ana.26852 (2023). 10.1002/ana.26852 38113326

[7] Huang, Y. *et al.* Risk factors associated with age at onset of Parkinson’s disease in the UK Biobank. *NPJ Parkinsons Dis.* **10**, 3. 10.1038/s41531-023-00623-9 (2024). 38167894 10.1038/s41531-023-00623-9 PMC10762149

[8] Jacobs, B. M. *et al.* Parkinson’s disease determinants, prediction and gene-environment interactions in the UK Biobank. *J. Neurol. Neurosurg. Psychiatry* **91**, 1046–1054. 10.1136/jnnp-2020-323646 (2020). 32934108 10.1136/jnnp-2020-323646 PMC7509524

[9] Paul, K. C., Schulz, J., Bronstein, J. M., Lill, C. M. & Ritz, B. R. Association of polygenic risk score with cognitive decline and motor progression in Parkinson disease. *JAMA Neurol.* **75**, 360–366. 10.1001/jamaneurol.2017.4206 (2018). 29340614 10.1001/jamaneurol.2017.4206 PMC5885856

[10] Pavelka, L. *et al.* Age at onset as stratifier in idiopathic Parkinson’s disease—effect of ageing and polygenic risk score on clinical phenotypes. *NPJ Parkinsons Dis.* **8**, 102. 10.1038/s41531-022-00342-7 (2022). 35945230 10.1038/s41531-022-00342-7 PMC9363416

[11] Escott-Price, V. *et al.* Polygenic risk of Parkinson disease is correlated with disease age at onset. *Ann. Neurol.* **77**, 582–591. 10.1002/ana.24335 (2015). 25773351 10.1002/ana.24335 PMC4737223

[12] Han, Y. *et al.* Genome-wide polygenic risk score identifies individuals at elevated Parkinson’s disease risk. *medRxiv*10.1101/2020.10.16.20212944 (2020).33336213

[13] Ibanez, L. *et al.* Parkinson disease polygenic risk score is associated with Parkinson disease status and age at onset but not with alpha-synuclein cerebrospinal fluid levels. *BMC Neurol.* **17**, 198. 10.1186/s12883-017-0978-z (2017). 29141588 10.1186/s12883-017-0978-z PMC5688622

[14] Elbaz, A. *et al.* CYP2D6 polymorphism, pesticide exposure, and Parkinson’s disease. *Ann. Neurol.* **55**, 430–434. 10.1002/ana.20051 (2004). 14991823 10.1002/ana.20051

[15] Fitzmaurice, A. G., Rhodes, S. L., Cockburn, M., Ritz, B. & Bronstein, J. M. Aldehyde dehydrogenase variation enhances effect of pesticides associated with Parkinson disease. *Neurology* **82**, 419–426. 10.1212/WNL.0000000000000083 (2014). 24491970 10.1212/WNL.0000000000000083 PMC3917685

[16] Gamache, P. L. *et al.* Exposure to pesticides and welding hastens the age-at-onset of Parkinson’s disease. *Can. J. Neurol. Sci.* **46**, 711–716. 10.1017/cjn.2019.248 (2019). 31342891 10.1017/cjn.2019.248

[17] Ratner, M. H., Farb, D. H., Ozer, J., Feldman, R. G. & Durso, R. Younger age at onset of sporadic Parkinson’s disease among subjects occupationally exposed to metals and pesticides. *Interdiscip. Toxicol.* **7**, 123–133. 10.2478/intox-2014-0017 (2014). 26109889 10.2478/intox-2014-0017 PMC4434105

[18] Jafari, S., Etminan, M., Aminzadeh, F. & Samii, A. Head injury and risk of Parkinson disease: A systematic review and meta-analysis. *Mov. Disord.* **28**, 1222–1229. 10.1002/mds.25458 (2013). 23609436 10.1002/mds.25458

[19] Aune, D. *et al.* Diabetes mellitus, prediabetes and the risk of Parkinson’s disease: A systematic review and meta-analysis of 15 cohort studies with 29.9 million participants and 86,345 cases. *Eur. J. Epidemiol.* **38**, 591–604. 10.1007/s10654-023-00970-0 (2023). 37185794 10.1007/s10654-023-00970-0 PMC10232631

[20] Grover, S. *et al.* Risky behaviors and Parkinson disease: A mendelian randomization study. *Neurology* **93**, e1412–e1424. 10.1212/WNL.0000000000008245 (2019). 31527283 10.1212/WNL.0000000000008245 PMC7010323

[21] Larsson, S. C. & Burgess, S. Appraising the causal role of smoking in multiple diseases: A systematic review and meta-analysis of Mendelian randomization studies. *EBioMedicine* **82**, 104154. 10.1016/j.ebiom.2022.104154 (2022). 35816897 10.1016/j.ebiom.2022.104154 PMC9278068

[22] Noyce, A. J. *et al.* Meta-analysis of early nonmotor features and risk factors for Parkinson disease. *Ann. Neurol.* **72**, 893–901. 10.1002/ana.23687 (2012). 23071076 10.1002/ana.23687 PMC3556649

[23] Fan, B. *et al.* What and how can physical activity prevention function on Parkinson’s disease?. *Oxid. Med. Cell. Longev.* **2020**, 4293071. 10.1155/2020/4293071 (2020). 32215173 10.1155/2020/4293071 PMC7042542

[24] Casper, D., Yaparpalvi, U., Rempel, N. & Werner, P. Ibuprofen protects dopaminergic neurons against glutamate toxicity in vitro. *Neurosci. Lett.* **289**, 201–204. 10.1016/s0304-3940(00)01294-5 (2000). 10961664 10.1016/s0304-3940(00)01294-5

[25] Marras, C., Canning, C. G. & Goldman, S. M. Environment, lifestyle, and Parkinson’s disease: Implications for prevention in the next decade. *Mov. Disord.* **34**, 801–811. 10.1002/mds.27720 (2019). 31091353 10.1002/mds.27720

[26] Delamarre, A. & Meissner, W. G. Epidemiology, environmental risk factors and genetics of Parkinson’s disease. *Presse Med.* **46**, 175–181. 10.1016/j.lpm.2017.01.001 (2017). 28189372 10.1016/j.lpm.2017.01.001

[27] Mappin-Kasirer, B. *et al.* Tobacco smoking and the risk of Parkinson disease: A 65-year follow-up of 30,000 male British doctors. *Neurology* **94**, e2132–e2138. 10.1212/WNL.0000000000009437 (2020). 32371450 10.1212/WNL.0000000000009437 PMC7526668

[28] Wahner, A. D., Bronstein, J. M., Bordelon, Y. M. & Ritz, B. Nonsteroidal anti-inflammatory drugs may protect against Parkinson disease. *Neurology* **69**, 1836–1842. 10.1212/01.wnl.0000279519.99344.ad (2007). 17984451 10.1212/01.wnl.0000279519.99344.ad

[29] Heilbron, K. *et al.* Unhealthy behaviours and risk of Parkinson’s disease: A mendelian randomisation study. *J. Parkinsons Dis.* **11**, 1981–1993. 10.3233/JPD-202487 (2021). 34275906 10.3233/JPD-202487 PMC8609708

[30] Hong, C. T., Chan, L. & Bai, C. H. The effect of caffeine on the risk and progression of Parkinson’s disease: A meta-analysis. *Nutrients*10.3390/nu12061860 (2020). 32580456 10.3390/nu12061860 PMC7353179

[31] Domenighetti, C. *et al.* Mendelian randomisation study of smoking, alcohol, and coffee drinking in relation to Parkinson’s disease. *J. Parkinsons Dis.* **12**, 267–282. 10.3233/JPD-212851 (2022). 34633332 10.3233/JPD-212851 PMC9211765

[32] Gabbert, C. *et al.* Coffee, smoking and aspirin are associated with age at onset in idiopathic Parkinson’s disease. *J. Neurol.* **269**, 4195–4203. 10.1007/s00415-022-11041-x (2022). 35235000 10.1007/s00415-022-11041-x PMC9294004

[33] Hamza, T. H. *et al.* Genome-wide gene-environment study identifies glutamate receptor gene GRIN2A as a Parkinson’s disease modifier gene via interaction with coffee. *PLoS Genet.* **7**, e1002237. 10.1371/journal.pgen.1002237 (2011). 21876681 10.1371/journal.pgen.1002237 PMC3158052

[34] Chuang, Y. H. *et al.* Gene-environment interaction in Parkinson’s disease: Coffee, ADORA2A, and CYP1A2. *Neuroepidemiology* **47**, 192–200. 10.1159/000450855 (2016). 28135712 10.1159/000450855 PMC5465963

[35] Lee, P. C. *et al.* Smoking and Parkinson disease: Evidence for gene-by-smoking interactions. *Neurology* **90**, e583–e592. 10.1212/WNL.0000000000004953 (2018). 29352099 10.1212/WNL.0000000000004953 PMC5818012

[36] Chuang, Y. H. *et al.* Pooled analysis of the HLA-DRB1 by smoking interaction in Parkinson disease. *Ann. Neurol.* **82**, 655–664. 10.1002/ana.25065 (2017). 28981958 10.1002/ana.25065 PMC5798887

[37] Luth, T. *et al.* Age at onset of LRRK2 p.Gly2019Ser is related to environmental and lifestyle factors. *Mov. Disord.* **35**, 1854–1858. 10.1002/mds.28238 (2020). 32875616 10.1002/mds.28238

[38] Wijeyekoon, R. *et al.* Associations between lifestyle factors and Parkinson’s Disease in an urban Sri Lankan clinic study. *Int. Arch. Med.*10.3823/2516 (2017). 29057010 10.3823/2516 PMC5646647

[39] Wilk, J. B. & Lash, T. L. Risk factor studies of age-at-onset in a sample ascertained for Parkinson disease affected sibling pairs: A cautionary tale. *Emerg. Themes Epidemiol.* **4**, 1. 10.1186/1742-7622-4-1 (2007). 17408493 10.1186/1742-7622-4-1 PMC1855322

[40] Yahalom, G. *et al.* Age at onset of Parkinson’s disease among Ashkenazi Jewish patients: Contribution of environmental factors, LRRK2 p.G2019S and GBA p.N370S mutations. *J. Parkinsons Dis.* **10**, 1123–1132. 10.3233/JPD-191829 (2020). 32310186 10.3233/JPD-191829

[41] De Reuck, J., De Weweire, M., Van Maele, G. & Santens, P. Comparison of age of onset and development of motor complications between smokers and non-smokers in Parkinson’s disease. *J. Neurol. Sci.* **231**, 35–39. 10.1016/j.jns.2004.12.003 (2005). 15792818 10.1016/j.jns.2004.12.003

[42] Gigante, A. F., Martino, T., Iliceto, G. & Defazio, G. Smoking and age-at-onset of both motor and non-motor symptoms in Parkinson’s disease. *Parkinsonism Relat. Disord.* **45**, 94–96. 10.1016/j.parkreldis.2017.09.022 (2017). 28988683 10.1016/j.parkreldis.2017.09.022

[43] Gigante, A. F. *et al.* Chronic coffee consumption and striatal DAT-SPECT findings in Parkinson’s disease. *Neurol. Sci.* **39**, 551–555. 10.1007/s10072-018-3253-1 (2018). 29362953 10.1007/s10072-018-3253-1

[44] Rosas, I. *et al.* Smoking is associated with age at disease onset in Parkinson’s disease. *Parkinsonism Relat. Disord.* **97**, 79–83. 10.1016/j.parkreldis.2022.03.005 (2022). 35364453 10.1016/j.parkreldis.2022.03.005

[45] Kang, U. J. *et al.* The BioFIND study: Characteristics of a clinically typical Parkinson’s disease biomarker cohort. *Mov. Disord.* **31**, 924–932. 10.1002/mds.26613 (2016). 27113479 10.1002/mds.26613 PMC5021110

[46] Mohammadi, D. The Harvard biomarker study’s big plan. *Lancet Neurol.* **12**, 739–740. 10.1016/S1474-4422(13)70155-8 (2013). 23809962 10.1016/S1474-4422(13)70155-8

[47] Chia, R. *et al.* Genome sequencing analysis identifies new loci associated with Lewy body dementia and provides insights into its genetic architecture. *Nat. Genet.* **53**, 294–303. 10.1038/s41588-021-00785-3 (2021). 33589841 10.1038/s41588-021-00785-3 PMC7946812

[48] Marek, K. *et al.* The Parkinson’s progression markers initiative (PPMI)—establishing a PD biomarker cohort. *Ann. Clin. Transl. Neurol.* **5**, 1460–1477. 10.1002/acn3.644 (2018). 30564614 10.1002/acn3.644 PMC6292383

[49] Holloway, R. *et al.* A phase 3 study of isradipine as a disease modifying agent in patients with early Parkinson disease(STEADY-PD III): Baseline characteristics and study update. *Mov. Disord.* **33**, S150–S150 (2018).

[50] Smolensky, L. *et al.* Fox Insight collects online, longitudinal patient-reported outcomes and genetic data on Parkinson’s disease. *Sci. Data* **7**, 67. 10.1038/s41597-020-0401-2 (2020). 32094335 10.1038/s41597-020-0401-2 PMC7039948

[51] Iwaki, H. *et al.* Accelerating medicines partnership: Parkinson’s disease. Genetic resource. *Mov. Disord.* **36**, 1795–1804. 10.1002/mds.28549 (2021). 33960523 10.1002/mds.28549 PMC8453903

[52] Purcell, S. *et al.* PLINK: A tool set for whole-genome association and population-based linkage analyses. *Am. J. Hum. Genet.* **81**, 559–575. 10.1086/519795 (2007). 17701901 10.1086/519795 PMC1950838

[53] Arnold, M., Raffler, J., Pfeufer, A., Suhre, K. & Kastenmuller, G. SNiPA: An interactive, genetic variant-centered annotation browser. *Bioinformatics* **31**, 1334–1336. 10.1093/bioinformatics/btu779 (2015). 25431330 10.1093/bioinformatics/btu779 PMC4393511

[54] Robin, X. *et al.* pROC: An open-source package for R and S+ to analyze and compare ROC curves. *BMC Bioinform.* **12**, 77. 10.1186/1471-2105-12-77 (2011). 10.1186/1471-2105-12-77 PMC3068975 21414208

[55] Wickham, H. *Use R!, 1 online resource (XVI, 260 pages 232 illustrations, 140 illustrations in color* (Springer International Publishing, 2016).

[56] R Core Team. *R: A Language and Environment for Statistical Computing* (R Foundation for Statistical Computing, 2023).

[57] Checkoway, H. *et al.* Parkinson’s disease risks associated with cigarette smoking, alcohol consumption, and caffeine intake. *Am. J. Epidemiol.* **155**, 732–738. 10.1093/aje/155.8.732 (2002). 11943691 10.1093/aje/155.8.732

[58] Chen, H. *et al.* Nonsteroidal antiinflammatory drug use and the risk for Parkinson’s disease. *Ann. Neurol.* **58**, 963–967. 10.1002/ana.20682 (2005). 16240369 10.1002/ana.20682

[59] Poly, T. N., Islam, M. M. R., Yang, H. C. & Li, Y. J. Non-steroidal anti-inflammatory drugs and risk of Parkinson’s disease in the elderly population: A meta-analysis. *Eur. J. Clin. Pharmacol.* **75**, 99–108. 10.1007/s00228-018-2561-y (2019). 30280208 10.1007/s00228-018-2561-y

[60] Becker, C., Jick, S. S. & Meier, C. R. NSAID use and risk of Parkinson disease: A population-based case-control study. *Eur. J. Neurol.* **18**, 1336–1342. 10.1111/j.1468-1331.2011.03399.x (2011). 21457177 10.1111/j.1468-1331.2011.03399.x

[61] Al-Azayzih, A. *et al.* Nonsteroidal anti-inflammatory drugs utilization patterns and risk of adverse events due to drug-drug interactions among elderly patients: A study from Jordan. *Saudi Pharm. J.* **28**, 504–508. 10.1016/j.jsps.2020.03.001 (2020). 32273811 10.1016/j.jsps.2020.03.001 PMC7132832

[62] Liu, E. Y. *et al.* Use of preventive aspirin among older US adults with and without diabetes. *JAMA Netw. Open* **4**, e2112210. 10.1001/jamanetworkopen.2021.12210 (2021). 34152419 10.1001/jamanetworkopen.2021.12210 PMC8218072

[63] Williams, C. D. *et al.* Aspirin use among adults in the U.S.: Results of a national survey. *Am. J. Prev. Med.* **48**, 501–508. 10.1016/j.amepre.2014.11.005 (2015). 25891049 10.1016/j.amepre.2014.11.005

[64] Pajares, M., I. Rojo, A., Manda, G., Boscá, L. & Cuadrado, A. Inflammation in Parkinson’s disease: Mechanisms and therapeutic implications. *Cells*10.3390/cells9071687 (2020). 32674367 10.3390/cells9071687 PMC7408280

[65] Marogianni, C. *et al.* Neurodegeneration and inflammation-an interesting interplay in Parkinson’s disease. *Int. J. Mol. Sci.*10.3390/ijms21228421 (2020). 33182554 10.3390/ijms21228421 PMC7697354

[66] Thaler, A. *et al.* Mutations in GBA and LRRK2 are not associated with increased inflammatory markers. *J. Parkinsons Dis.* **11**, 1285–1296. 10.3233/JPD-212624 (2021). 33998549 10.3233/JPD-212624 PMC8461659

[67] Lüth, T. *et al.* Interaction of mitochondrial polygenic score and environmental factors in LRRK2 p.Gly2019Ser parkinsonism. *medRxiv*10.1101/2023.01.02.23284113 (2023). 37482924 10.1002/mds.29563

[68] Billingsley, K. J. *et al.* Mitochondria function associated genes contribute to Parkinson’s Disease risk and later age at onset. *NPJ Parkinsons Dis.* **5**, 8. 10.1038/s41531-019-0080-x (2019). 31123700 10.1038/s41531-019-0080-x PMC6531455

[69] Wojcik, G. L. *et al.* Genetic analyses of diverse populations improves discovery for complex traits. *Nature* **570**, 514–518. 10.1038/s41586-019-1310-4 (2019). 31217584 10.1038/s41586-019-1310-4 PMC6785182

[70] Sirugo, G., Williams, S. M. & Tishkoff, S. A. The missing diversity in human genetic studies. *Cell* **177**, 26–31. 10.1016/j.cell.2019.02.048 (2019). 30901543 10.1016/j.cell.2019.02.048 PMC7380073

[71] Caliebe, A. *et al.* Including diverse and admixed populations in genetic epidemiology research. *Genet. Epidemiol.* **46**, 347–371. 10.1002/gepi.22492 (2022). 35842778 10.1002/gepi.22492 PMC9452464

