# Supplementary material for: The combined effect of lifestyle factors and polygenic scores on age at onset in Parkinson’s disease
Source: Sci Rep. 2024 Jun 25;14:14670. doi: 10.1038/s41598-024-65640-x (PMC11199580; doi:10.1038/s41598-024-65640-x)
Supplement: Supplementary file 1 — Supplementary Information. [file 41598_2024_65640_MOESM1_ESM.pdf]

SUPPLEMENTARY MATERIAL FOR

The combined effect of lifestyle factors and polygenic scores on age at onset in Parkinson's disease

Carolin Gabbert[1], MSc, Leonie Blöbaum[1], BSc, Theresa Lüth[1], MSc, Prof. Inke R. König[2], Amke Caliebe[3], PhD, Sebastian Sendel[3], MSc, Björn-Hergen Laabs[2], PhD, Christine Klein[1], MD, Joanne Trinh[1*], PhD

Author affiliations:

[1]Institute of Neurogenetics, University of Lübeck, Lübeck, Germany

[2]Institute of Medical Biometry and Statistics, University of Lübeck, Lübeck, Germany

[3]Institute of Medical Informatics and Statistics, Kiel University, University Hospital Schleswig-Holstein, Kiel, Germany

[*]Correspondence to: Joanne Trinh

University of Lübeck

Ratzeburger Allee 160

23538 Lübeck, Germany

Email: joanne.trinh@uni-luebeck.de

Tel.: +49-451-31018202

SUPPLEMENTARY FIGURES

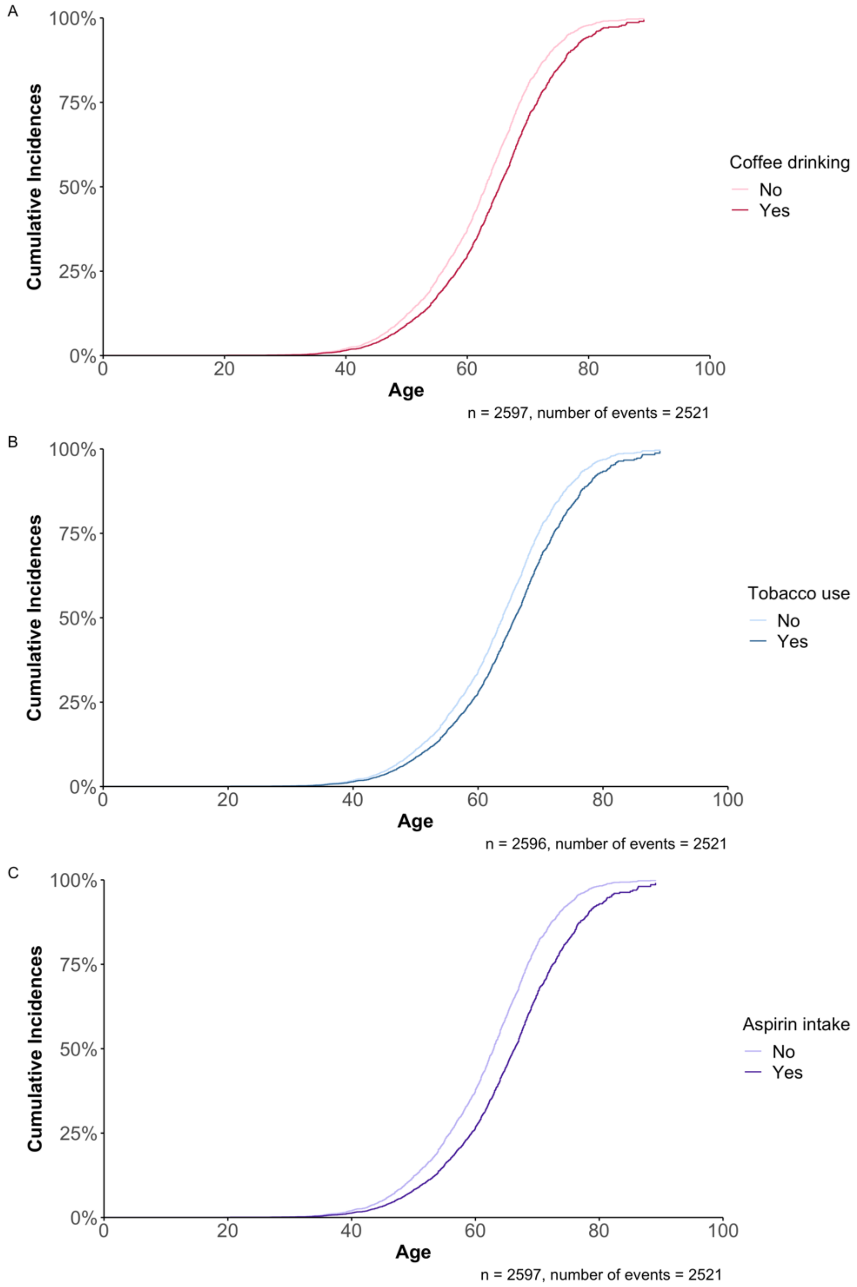

Supplementary Figure 1. Plot of the Cox proportional hazards models to investigate the association between the lifestyle factors coffee drinking, tobacco use, and aspirin intake on the AAO of PD patients, while censoring with the AAE of healthy controls.

(A) The different curves describe the coffee drinkers and non-coffee drinkers. A Cox proportional hazards model was used to investigate the difference in AAO while censoring with the AAE of healthy controls. The sex and the study site were additionally included as covariates (→ coxph(formula = Surv(AAO/AAE, Diagnosis) ~ Coffee + Sex + Study, data = data)) **(B)** The different curves describe the tobacco users and non-tobacco users. A Cox proportional hazards model was used to investigate the difference in AAO while censoring with the AAE of healthy controls. The sex and the study site were additionally included as covariates (→ coxph(formula = Surv(AAO/AAE, Diagnosis) ~ Tobacco + Sex + Study, data = data)) **(C)** The different curves describe the aspirin users and non-aspirin users. A Cox proportional hazards model was used to investigate the difference in AAO while censoring with the AAE of healthy controls. The sex and the study site were additionally included as covariates (→ coxph(formula = Surv(AAO/AAE, Diagnosis) ~ Aspirin + Sex + Study, data = data))

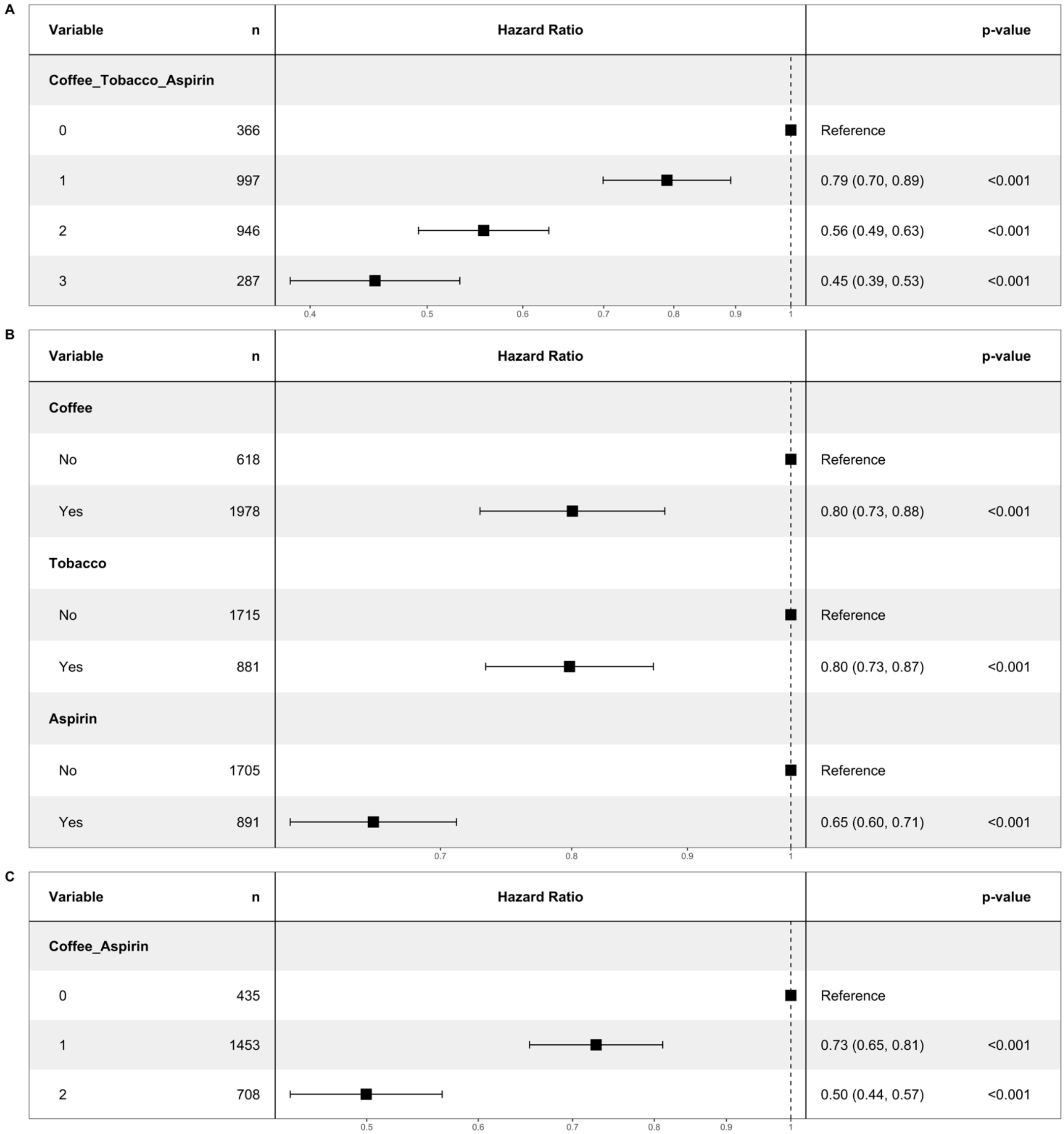

Supplementary Figure 2. Plot of the Cox proportional hazards model to investigate the additive effects between the use of the lifestyle factors coffee drinking, tobacco use, and aspirin intake on the AAO of PD patients, while censoring with the AAE of healthy controls.
(A) The use of the lifestyle factors coffee drinking, tobacco use, and aspirin intake was used as cumulative number (0-3). The sex and study site were additionally included as covariables but are not displayed. (→ coxph(formula = Surv(AAO/AAE, Diagnosis) ~ Coffee/Tobacco/Aspirin + Sex + Study, data = data)). **(B)** The lifestyle factors coffee drinking, tobacco use, and aspirin intake were included as separate covariables. The sex and study site were additionally included as covariables but are not displayed. (→ coxph(formula = Surv(AAO/AAE, Diagnosis) ~ Coffee + Tobacco + Aspirin + Sex + Study, data = data)). **(C)** The use of the lifestyle factors coffee drinking and aspirin intake was used as cumulative number (0-2). The sex and study site were additionally included as covariables but are not displayed. (→ coxph(formula = Surv(AAO/AAE, Diagnosis) ~ Coffee/Aspirin + Sex + Study, data = data)).

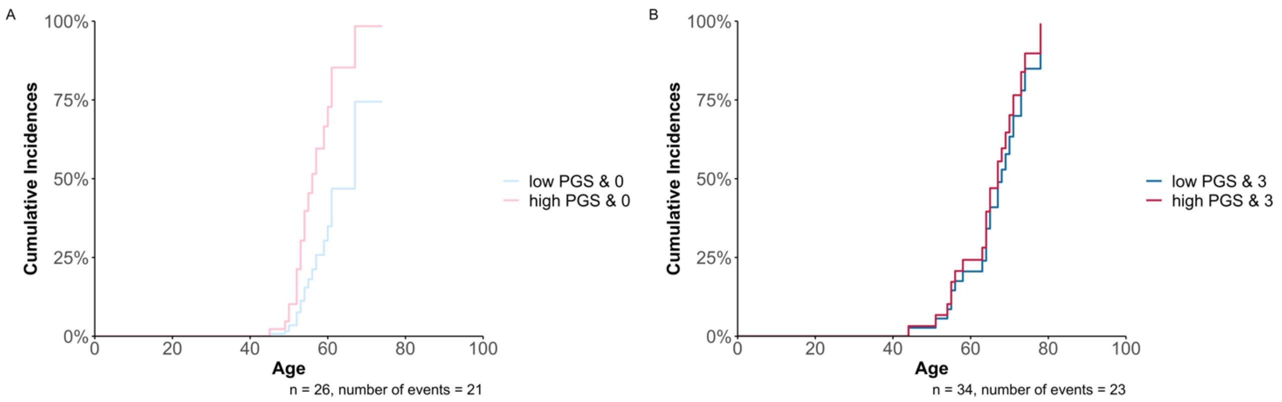

Supplementary Figure 3. Additive effects of the PGS and lifestyle factors on the AAO of PD patients, while censoring with the AAE of healthy controls.

(A) The different curves describe the PGS categorized into "low PGS" and "high PGS" according to the median PGS in the subgroup of participants that used no protective lifestyle factor. A Cox proportional hazards model was used to investigate the difference in AAO while censoring with the AAE of healthy controls. The sex and the first two PCs were additionally included (→ coxph(formula = Surv(AAO/AAE, Diagnosis) ~ PGS low/high + Sex + PC1 + PC2, data = data_Coffee/Tobacco/Aspirin 0)). **(B)** The different curves describe the PGS categorized into "low PGS" and "high PGS" according to the median PGS in the subgroup of participants that used all three protective lifestyle factors. A Cox proportional hazards model was used to investigate the difference in AAO while censoring with the AAE of healthy controls. The sex and the first two PCs were additionally included (→ coxph(formula = Surv(AAO/AAE, Diagnosis) ~ PGS low/high + Sex + PC1 + PC2, data = data_Coffee/Tobacco/Aspirin 3)).

SUPPLEMENTARY TABLES

Supplementary Table 1. Linear model on the association of coffee drinking, tobacco use, and aspirin intake with AAO in the *GBA1*-PD study group

	**Estimate**	**Standard error**	***p*-value**	
**Coffee drinking (binary) (*n* = 145)[1]**				
Intercept	58.8245	1.9240	**$<2x10^{-16}$**	*
Coffee drinking (binary)	3.3597	2.0773	0.1080	
Sex (Male)	0.5488	1.6176	0.7349	
**Coffee drinking dosage (*n* = 113)[1]**				
Intercept	60.7349	1.5662	**$<2x10^{-16}$**	*
Coffee drinking dosage	0.0791	0.1137	0.4878	
Sex (Male)	0.7757	1.7817	0.6641	
**Coffee drinking duration (*n* = 93)[1]**				
Intercept	55.2783	1.7340	**$<2x10^{-16}$**	*
Coffee drinking duration	0.2423	0.0455	**$7x10^{-7}$**	*
Sex (Male)	-0.4705	1.9440	0.8093	
**Tobacco use (binary) (*n* = 149)[1]**				
Intercept	60.2231	1.2026	**$<2x10^{-16}$**	*
Tobacco use (binary)	3.6527	1.5808	**0.0223**	*
Sex (Male)	0.9111	1.5198	0.5498	
**Tobacco use dosage (*n* = 124)[1]**				
Intercept	60.8739	1.2053	**$<2x10^{-16}$**	*
Tobacco use dosage	0.1386	0.0791	0.0823	
Sex (Male)	-0.2229	1.6733	0.8942	
**Tobacco use duration (*n* = 121)[1]**				
Intercept	60.7810	1.2286	**$<2x10^{-16}$**	*
Tobacco use duration	0.2193	0.0882	**0.0143**	*
Sex (Male)	0.3614	1.6704	0.8291	
**Aspirin intake (binary) (*n* = 92)[1]**				
Intercept	59.3073	1.6076	**$<2x10^{-16}$**	*
Aspirin intake (binary)	3.5143	2.2708	0.1253	
Sex (Male)	1.1559	2.0902	0.5816	
**Aspirin intake dosage (*n* = 88)[1]**				
Intercept	59.3123	1.4826	**$<2x10^{-16}$**	*
Aspirin intake dosage	0.4355	0.2213	0.0524	
Sex (Male)	1.5660	2.0077	0.4376	
**Aspirin intake duration (*n* = 83)[1]**				
Intercept	58.985	1.5121	**$<2x10^{-16}$**	*
Aspirin intake duration	0.4810	0.2066	**0.0224**	*
Sex (Male)	1.7848	2.0705	0.3913	
**All lifestyle factors (binary) (*n* = 89)[2]**				
Intercept	56.4789	2.7708	**$<2x10^{-16}$**	*
Coffee drinking (binary)	0.2295	2.8641	0.9363	
Tobacco use (binary)	6.7844	2.1707	**0.0024**	*
Aspirin intake (binary)	2.1989	2.2613	0.3336	
Sex (Male)	1.8093	2.0733	0.3853	

[1]glm(formula = AAO ~ Lifestyle factor + Sex, family = gaussian, data=data).
[2]glm(formula = AAO ~ Coffee drinking (binary) + Tobacco use (binary) + Aspirin intake (binary) + Sex, family = gaussian, data=data).
* *p*-value < 0.05 are highlighted in bold.
Abbreviations: AAO, age at onset; PD, Parkinson's disease; glm, generalized linear model.

Supplementary Table 2. Linear model on the association of coffee drinking, tobacco use, and aspirin intake with AAO using *GBA1* mutation carrier status (*GBA1*-PD vs. iPD) as another covariate

	Estimate	Standard error	*p*-value	
**Coffee drinking (binary) (*n* = 2666)**[1]				
Intercept	57.4021	0.4039	**<2x10^{-16}**	*
Coffee drinking (binary)	2.9247	0.4293	**2x10^{-11}**	*
Sex (Male)	1.1880	0.3657	**0.0012**	*
GBA1 mutation status (*GBA1*-PD)	1.4501	0.8007	0.0702	
**Coffee drinking dosage (*n* = 2438)**[1]				
Intercept	58.7078	0.3216	**<2x10^{-16}**	*
Coffee drinking dosage	0.0815	0.0193	**2x10^{-5}**	*
Sex (Male)	1.0858	0.3880	**0.0052**	*
GBA1 mutation status (*GBA1*-PD)	2.0984	0.8989	**0.0197**	*
**Coffee drinking duration (*n* = 1816)**[1]				
Intercept	55.5061	0.3723	**<2x10^{-16}**	*
Coffee drinking duration	0.1791	0.0099	**<2x10^{-16}**	*
Sex (Male)	-0.4503	0.4184	0.2821	
GBA1 mutation status (*GBA1*-PD)	1.4076	0.9306	0.1306	
**Tobacco use (binary) (*n* = 2670)**[1]				
Intercept	58.8585	0.2913	**<2x10^{-16}**	*
Tobacco use (binary)	2.0021	0.3847	**2x10^{-7}**	*
Sex (Male)	1.3576	0.3645	**0.0002**	*
GBA1 mutation status (*GBA1*-PD)	1.7411	0.7919	**0.0280**	*
**Tobacco use dosage (*n* = 2410)**[1]				
Intercept	59.1459	0.2808	**<2x10^{-16}**	*
Tobacco use dosage	0.0681	0.0125	**6x10^{-8}**	*
Sex (Male)	1.0795	0.3832	**0.0049**	*
GBA1 mutation status (*GBA1*-PD)	1.4818	0.8588	0.0846	
**Tobacco use duration (*n* = 2180)**[1]				
Intercept	59.9896	0.2977	**<2x10^{-16}**	*
Tobacco use duration	0.1311	0.0221	**3x10^{-9}**	*
Sex (Male)	0.9518	0.4015	**0.0178**	*
GBA1 mutation status (*GBA1*-PD)	1.8386	0.8750	**0.0357**	*
**Aspirin intake (binary) (*n* = 2612)**[1]				
Intercept	58.2492	0.2813	**<2x10^{-16}**	*
Aspirin intake (binary)	4.6349	0.3854	**<2x10^{-16}**	*
Sex (Male)	0.7787	0.3656	**0.0333**	*
GBA1 mutation status (*GBA1*-PD)	0.9098	0.9778	0.3522	
**Aspirin intake dosage (*n* = 2495)**[1]				
Intercept	58.8474	0.2811	**<2x10^{-16}**	*
Aspirin intake dosage	0.2684	0.0383	**3x10^{-12}**	*
Sex (Male)	1.0059	0.3757	**0.0075**	*
GBA1 mutation status (*GBA1*-PD)	1.1040	1.0053	0.2722	
**Aspirin intake duration (*n* = 2166)**[1]				
Intercept	58.6431	0.2888	**<2x10^{-16}**	*
Aspirin intake duration	0.2948	0.0285	**<2x10^{-16}**	*
Sex (Male)	0.5388	0.3982	0.1762	
GBA1 mutation status (*GBA1*-PD)	1.3935	1.0246	0.1739	
**All lifestyle factors (binary) (*n* = 2610)**[2]				
Intercept	56.1502	0.4099	**<2x10^{-16}**	*
Coffee drinking (binary)	2.3116	0.4313	**9x10^{-8}**	*
Tobacco use (binary)	1.5787	0.3858	**4x10^{-5}**	*
Aspirin intake (binary)	4.5174	0.3820	**<2x10^{-16}**	*
Sex (Male)	0.4930	0.3643	0.1761	
GBA1 mutation status (*GBA1*-PD)	0.5397	0.9842	0.5835	

[1]glm(formula = AAO ~ Lifestyle factor + Sex + *GBA1* mutation status, family = gaussian, data=data).
[2]glm(formula = AAO ~ Coffee drinking (binary) + Tobacco use (binary) + Aspirin intake (binary) + Sex + *GBA1* mutation status, family = gaussian, data=data).
* p-value < 0.05 are highlighted in bold.
Abbreviations: AAO, age at onset; PD, Parkinson's disease; glm, generalized linear model.
